# Advancing good governance in data sharing and biobanking - international aspects

**DOI:** 10.12688/wellcomeopenres.15540.1

**Published:** 2019-11-22

**Authors:** Buddhika Fernando, Mandella King, Athula Sumathipala

**Affiliations:** 1 Institute for Research and Development, 393/3 Lily Avenue, Robert Gunawardena Mawatha, Battaramulla, 10120, Sri Lanka; 2 St. Joseph’s Catholic Hospital, Monrovia, Liberia; 3 School of Primary, Community and Social Care, Faculty of Medicine, Keele University, Keele, Staffordshire, ST5 5BG, UK

**Keywords:** Data-sharing, biobanking, data governance frameworks, LMIC, capacity building, collaboration, fairness

## Abstract

Ethical and effective data-sharing among countries can be achieved by considering the interests of all relevant parties: research participants, researchers and funders. Fears of exploitation, however, both of research participants and researchers from low- and middle-income countries (LMIC), can undermine the free flow of data necessary for scientific advancement.

In this Open Letter, two case studies presented at the 2018 Global Forum on Bioethics in Research meeting on the Ethics of data sharing and biobanking in Cape Town, South Africa, function as the focal point for a reflection on the attributes of an ideal model of good data governance and how it can help support ethical best practices in biobanking and data sharing.

Consideration of the case studies as well as the literature indicate three broad principles that need to be reflected in an ideal data governance framework: (i) collaboration - both among researchers as well as between researchers and participants, (ii) fairness – ensuring that all parties in international collaborations, the data provider, primary data gathering LMIC researcher and the high income country (HIC) institution/funder are treated fairly, and (iii) working towards a level playing field – neither collaboration nor fairness can be effectively achieved with the existing power differential between HIC and LMIC researchers/institutions; it is therefore necessary to work towards achieving a more level playing field between partners in research collaborations.

Promoting good governance of data through fair, efficient and accountable governance frameworks can help build trust and ensure continued international data sharing.

## Disclaimer

The views expressed in this article are those of the author(s). Publication in Wellcome Open Research does not imply endorsement by Wellcome.

## Background

**Figure 1.**
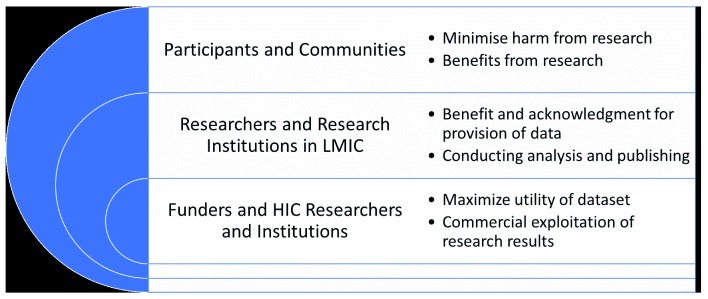
Good governance requires consideration of benefits and interests of all parties related to research.

International data sharing is garnering greater attention as it is now well-established that obstructing the free flow of data is an important factor that can threaten global health and global security, in addition to impeding the obvious benefits of data sharing in healthcare research. The importance of data sharing, the ethical imperative to share data and the criteria required for such data sharing to be ethical have been frequently discussed and well-established ( Bull *et al.*, 2015; Pisani & AbouZahr, 2010; Singh & Shetty, 2017). The literature shows almost unanimous agreement on the need to show respect for the data-providing research participants and their communities through seeking their informed consent for the use of their data, engaging the communities in the work and by ensuring adequate protection against any potential harm they may suffer as a result of providing data and/or samples ( Aitken *et al.*, 2016; Parker & Bull, 2015). It is equally necessary to consider the benefits and interests of all parties relevant to data provision and analysis to ensure ethical data sharing and to incentivize data sharing ( Figure 1). The term data is used here in a broad sense, encompassing primary and secondary data as well as samples that are ultimately converted to data.

To operationalize these considerations, they need to be incorporated into ethically appropriate governance frameworks and implemented by ethics review boards and access committees through access review procedures and oversight processes to determine who can have access to data and samples for what purposes.

However, the procedures for review and oversight of data and sample access are not sufficiently organized to achieve the full benefits of data and sample sharing ( Tiffin *et al.*, 2019; van Panhuis *et al.*, 2014), particularly concerning international transactions of data and samples. Many attempts are being made to implement governance frameworks that are globally applicable, as well as safeguard the interests and address the concerns of all relevant parties. Such an effective framework needs to be fair, efficient, show accountability and, just as importantly, be sustainable in the long run ( de Vries *et al.*, 2017; Klingstrom *et al.*, 2016).

Setting up and managing a biobank or a database effectively requires extensive laboratory infrastructure, state-of-the-art technology and expertise in the collection, storage and analysis of data and samples. Although initial funding for setting up a biobank is more easily available, long-term funding for maintenance of the biobank and data analysis is much scarcer, even in high-income countries (HICs) ( Doucet *et al.*, 2017). Two solutions have been proposed to deal with this issue of lack of infrastructure, technology, expertise and funding for data storage and analysis in low- and middle-income country (LMIC) settings: a commercialization model where samples and data are monetized, and secondly, to transfer samples and data to HICs where the capacity and infrastructure to store and analyse already exists, whilst ensuring access and benefit to the data/sample providers from LMIC ( Bubela *et al.*, 2015; Doucet *et al.*, 2017; Mulder *et al.*, 2017).

The latter situation of data and sample transfer is what happens most commonly, though providing access and benefit does not necessarily take place. Although data is targeted to be open access, data and sample flow is almost always unidirectional, from LMICs to HICs ( Serwadda *et al.*, 2018). Deepening concerns about exploitation, fairness and access to benefits from data have ultimately led to severe impediments to critically important data sharing between countries ( Caulfield *et al.*, 2014; Sedyaningsih *et al.*, 2008). The concerns regarding governance of international data sharing revolve around several different aspects of this fear of exploitation.

Firstly, LMICs are concerned that the samples and data taken from pathogens found in their countries are taken and used without their consent to develop vaccines and medicines that are later priced at levels unaffordable to LMIC countries, preventing the very people who contributed to the treatment development from having access to the treatment ( Sedyaningsih *et al.*, 2008). This is the issue that Indonesia raised in 2007, stating that the H5N1 samples sent from Indonesia to the World Health Organisation (WHO) were transferred to commercial pharmaceutical manufacturers without Indonesia’s consent; a vaccine subsequently developed was not affordable or available to citizens of the originating countries such as Indonesia and Thailand ( Hong, 2018). More recently, there were widely reported incidents of scientists from the countries of origin of viral samples being unable to access the samples exported to laboratories in developed countries ( Telegraph, 2019). These samples, which remain linked to the patients’ personal data, were admittedly taken and exported to these countries without consent for research from the patients. There are several other incidences of countries refusing permission to share viral samples – both during the Ebola epidemic and the Yellow Fever epidemic in 2016. ( Abramowitz *et al.*, 2018).

A second aspect of these concerns regarding exploitation is that researchers and research institutions from LMICs do not receive the benefits from their initial work in the form of their ability to continue the research, attributions and publications ( Hajduk *et al.*, 2019). Due to insufficient research infrastructure (technological and human) in LMICs, institutions and researchers in well-resourced countries can analyse and publish data much faster, resulting in lack of reward (in the form of recognition of their efforts and lack of publications, extending to lack of career progression) for LMIC scientists who bear the initial risk and burden of collecting data/samples. For example, in the H5N1 case, other countries published and presented data analyses of Indonesian virus samples without reasonable notification, permission, opportunities for collaboration on further research or attribution to the scientists generating and sharing the data/samples ( Sedyaningsih *et al.*, 2008). Furthermore, there is a risk of misinterpretation and misuse of data when researchers without any connections to or understanding of the data-supplying communities engage in data analysis. There is also the concern of potential violations of privacy and patient re-identification, especially in cases where patients’ personal data remain linked to the samples/data ( Pisani & AbouZahr, 2010; Rani & Buckley, 2012).

The case studies below discuss two situations where international data sharing caused concerns. The first case discusses practical experience of how these concerns were effectively managed and the second discusses the results of a qualitative study that investigated the underlying causes behind such concerns.

## Case study 1: 20 years of ethical challenges in setting up and maintaining a twin registry and biobank in Sri Lanka

The Sri Lanka Twin Registry was set up in 1997 as the first twin register in South Asia, by the Institute for Research and Development (IRD), Sri Lanka, with funding from the Wellcome Trust. It is still one of the very few large-scale, functional, population-based twin registries in a LMIC with 9,570 twin pairs and 46 triplets from the Colombo district of Sri Lanka, and a separate island-wide volunteer register comprising 7,000 twin pairs and 86 triplets. The first wave of research using the register was the Colombo Twin and Singleton Study (COTASS) in 2005, conducted to estimate prevalence, heritability and gene-environmental interplay of a range of psychiatric disorders (depression, somatisation, post-traumatic stress disorder, and substance abuse), resulting in 18 publications and another eight papers from the twin research consortium, the CODATwins Project, which pooled data from 67 worldwide twin projects. A follow-up study of the same cohort was completed in 2016, examining the prevalence and inter-relationship of key cardiovascular/metabolic risk markers and diabetes with depression and anxiety. The Sri Lankan Twin Registry Biobank was established in 2012 as a major component of the second wave data collection of COTASS. The biobank consists of 3,483 serum and 3,360 DNA samples of twins and singletons and is the first twin bio-specimen biobank in South Asia. Two papers have been published using the follow-up data and two papers submitted, while more are planned. In 2018, the Medical Research Council (MRC), UK, awarded a pump-priming grant to set up an infant, child and adolescent twin registry for mental health research. Ethical sensitivities are similar in all projects, arising from the research focus including a vulnerable group (minors, some having mental illness), involving proxy consent, potential for complex genetic and omics research, sensitive personal data and most of all, the complications related to international collaborations.

One of the first hurdles the IRD faced when setting up the twin registry was the unfounded accusations of collecting and exporting genetic information of Sri Lankan twins ( Daily Mirror Sri Lanka, 2002). The response from the IRD was to engage in extensive awareness-raising and community engagement activities using multiple routes, ranging from regular newspaper advertisements, feature articles, radio talks, exhibitions, leisure activities and television programmes to the usual small group discussions and focus groups, as well as sensitising other professional and academic groups. The tri-lingual magazine of the IRD, ‘Gaveshana’ (Explorations), mainly aimed at school children and undergraduates as well as the wider general public, published thematic issues on twin research, ethics and governance. Cultural activities engaging twins, publication of a newsletter in the local languages called ‘Twin News’, regularly updated and distributed among the members of the twin registry, formed part of the community engagement activities. 

The lack of ethical guidelines and a governance framework that was fit for purpose was another key issue the IRD faced at the time of setting up the twin registry. The response of the IRD was to initiate standard-setting, based on a blend of the existing guidelines and the customs, social and moral norms of the Sri Lankan culture. The IRD developed and published research ethics guidelines titled ‘Research Ethics from a Developing World Perspective’ with the help of many Sri Lankan academics and researchers as well as input from world-leading ethics experts. The IRD engaged in extensive work in ethics capacity building under the theme ‘Ethics: A friend of Research’ and carried out many Wellcome-funded ethics training courses at both basic and advanced levels, earning a reputation that later led to the UNESCO Ethics capacity building programs being delivered in Sri Lanka collaboratively with the IRD (2017).

Due to the lack of infrastructure and technical expertise in Sri Lanka, it was suggested that the biological samples from the twin biobank should be sent abroad for genetic analysis. The IRD team chose to delay the genetic analysis and, although it took five years, necessary infrastructure including a genetic laboratory as well as research expertise were developed at the IRD with extensive support from the Wellcome Trust and international collaborators. The decision to delay the genetic work until the infrastructure and expertise were developed and the IRD was confident of its ability to effectively manage the multiple ethical and technical challenges raised by this work has, in retrospect, showed itself to be the correct one, since the IRD genetic laboratory is now carrying out extensive work in genetic analysis. Even more importantly, the capacity building programs carried out by the IRD in this sphere help build expertise, not only in Sri Lanka, but the entire South Asian region.

### Critical reflection

As the twin registry was set up at a time when research ethics was at a nascent stage of being codified in Sri Lanka, the IRD team faced multiple challenges in ensuring that the ethical concerns specific to building a database in an LMIC were addressed. As the project progressed, the twin database expanded into a biobank, bringing about further challenges related to the collection, storage, use and protection of samples. The lack of consensus on a broad ethical framework and overarching ethical guidance was also a key issue at the time. This was complicated by the lack of effective guidance on managing relationships with influential international research collaborations, in a manner that was respectful of cultural sensitivities of the research participants/communities, ensured research benefits were shared equitably and at the same time ensured the generation of maximum utility from the research funds.

The negative media campaign that was framed as a warning from medical experts sensationalized cultural sensitivities and concerns founded on past misdemeanours by other researchers and historical injustices. The IRD recognised that the concerns, though unfounded in this situation, were valid expressions of the mistrust the community had towards unethical researchers and therefore aimed to achieve true community engagement through developing mutual respect and trust to combat misinformation. The activities carried out helped build understanding and awareness about the research and helped the twins and wider society to understand that the research team viewed them as participants with an important role to play in an activity that would benefit humankind. This feeling of mutual respect and camaraderie between the research team and the participants was the key factor that helped the IRD team overcome negative publicity and carry out two waves of research among these twins since 1997. 

Existing ethical guidelines and frameworks were reflected on and found to address issues that dominate ethics in a Western context and rarely address real life issues faced by LMIC researchers. For example, the case studies usually provided are rarely applicable in the developing world context, and the mechanisms suggested to address ethical issues are not feasible in the extremely resource-constrained situations faced in LMICs. It is therefore necessary to initiate home-grown mechanisms to deal with these constraints and develop ethical guidelines and governance frameworks to support LMIC researchers. 

The IRD considers that its international research collaborators for the twin registry and biobank-related projects exemplified what research collaborations between the global north and south should aim for: being generous in sharing expertise, carrying out extensive capacity building efforts, supporting Sri Lankan researchers by enabling first authorship of publications and, most of all, being respectful of cultural sensitivities. Their support enabled research capacity building not only at the IRD but in the wider Sri Lankan research community through numerous training programs. Within this context, the IRD had to seriously consider the request to transfer biological samples to the UK for analysis based on the premise that it would take time to develop the necessary infrastructure and expertise in Sri Lanka. It was necessary to weigh the benefits of faster research output by transferring samples abroad against the detrimental impact of Sri Lankan researchers being relegated to mere sample gatherers and losing the long-term benefits of developing capacity in Sri Lanka.

## Case study 2: Rumours and fears endanger feasibility of biobanking in Liberia

During the Ebola outbreak, many Liberian patients with infectious diseases other than Ebola saw their healthcare needs neglected. Malaria became one such rather neglected disease and the Saint Joseph’s Catholic Hospital (SJCH) undertook a number of measures to support the development of new diagnostic, preventive and elimination tools for malaria, as well as to enhance local capacity building in malaria research in Liberia. In this context, a study was carried out to investigate the feasibility of establishing a SJCH-hosted biorepository of blood samples obtained from malaria-exposed individuals attending the hospital that aimed: i) to support the development of improved cost-efficacious high-sensitive malaria diagnostic tools; ii) to support quality assessment of presumably substandard and unregistered rapid diagnostic tests that are known to be easily available over-the-counter in Liberia, and iii) to provide information on the burden of malaria and antimalarial resistance to guide public health interventions. This study was nested within a mixed-methods study that aimed to assess the burden of malaria among pregnant women attending antenatal care at the SJCH ( MESA Malaria, 2018); the qualitative research component of the study explored pregnant women’s, traditional leaders’ and health personnel’s perspectives on barriers and opportunities for pregnant women to consent to participate in malaria research.

To inform the design of the study and to plan dissemination at community-level, a group of ten traditional leaders received training in medical research ethics and were invited to constitute a Community Advisory Board. Qualitative research carried out among this group explored the drivers of acceptability to engage in research that may involve collection, transport, storage, and use of blood samples, i.e. the factors that could directly impact the successful establishment and operation of the biobank of blood samples. The study revealed that the concerns centred primarily around three themes.

Fear of discrimination and restricted access to healthcare: A key factor that can impede consent for biobanking is the widespread fear that samples collected in clinical and research settings may be used to test for stigmatizing diseases such as HIV without seeking permission from the sample donor. On the other hand, given the difficulties of accessing free-of-charge healthcare services, fear that refusal to provide samples could restrict their access to healthcare, as well as therapeutic misconception, can motivate people to consent to participation in biobanking.

Cultural issues related to lack of trust and fear of exploitation: Any activity involving blood is considered to be of a sensitive nature in the Liberian culture. Blood sample extraction in a clinical setting is acceptable to most, whereas the same activity in a non-clinical setting would be viewed with suspicion. This was exacerbated in some cases by exposure to Ebola vaccine trials in Monrovia, leading to the belief that samples are collected from trial subjects for illicit purposes. The generalised lack of trust in the healthcare and research establishment impedes voluntary participation in biobanking activities. Closely related to the issue of lack of trust in healthcare service providers and researchers, there are widespread concerns that researchers would export the samples collected for financial benefit without sharing such benefits with the researched communities.

Lack of accountability and good governance: Communities are aware that during the Ebola epidemics, there were various initiatives to collect samples that were subsequently shipped abroad for research or public health purposes (i.e. Ebola vaccine trials, Ebola Treatment Centers, Plasmapheresis Unit). Communities invited to assent to biobanking in Liberia may perceive that the destination and usage of exported samples cannot be controlled by local research staff and, hence, that fair conditions for de-identification and re-identification, usage, protection of personal data, storage, retrieval, tracking and disposal of samples cannot be guaranteed to the study participants.

### Critical reflection

Fear of discrimination and of exploitation, exacerbated by lack of trust in the healthcare system, is the single factor that can undermine effective biobanking efforts in Liberia. To help re-establish lost trust and for ethical, fair and successful biobanking, it is necessary to engage participants and communities by giving them information, empowering them with greater decision-making over the use of their data/samples, setting up clear mechanisms for accountability and good governance and lastly and most importantly, implementing fair benefit sharing plans.

Innovative approaches to improve trust, targeted at both individuals and communities, need to be designed and implemented ahead of the creation of biobanks. Clear information on risks, benefits and compensation, as well as reassurance that there will be no negative implications if consent is withheld, should be provided during the consent process to all approached individuals to avoid undue inducement to participate. Broad consent may be inappropriate in these cases as people may want to know the exact intended use of their samples and may want reassurance that there will be no negative consequences from participation. During this study, it was suggested that implementing a stepped wedge consent system could be used so that participants can decide exactly what clinical, public health and research use of their data and samples they authorize, as well as to what type of communication from the biobank regarding the use, export, retrieval and disposal of their samples they expect to receive. Given the cost and the administrative difficulties, it is not feasible to use a stepped wedge consent system in a large research population; therefore, a generalised approach to sample and data usage could potentially be based on people’s preferences derived from this project. 

Sustainability of a biobank in Liberia could largely depend on cost recovery of its running costs. Study participants’ narratives indicate that community members may not oppose the commercialization of their samples provided a benefit sharing plan engaging participants and their communities is implemented. However, the feasibility of a pay-per-service system should be evaluated considering the adverse impact it may have on the perception of trustworthiness. To improve trustworthiness, a clear structure for accountability and a benefit sharing plan needs to be agreed upon by research, regulatory, hospital staff and the affected communities at the initiation of a project. In addition to improving communication on research plans and proposed activities, all stakeholders need to receive accurate information on accountability, human resources, financial and sustainability issues. A transparent attitude and willingness to negotiate benefit sharing may help communities trust in biobanking research in Liberia.

## Discussion and conclusions

Refusal to share data and samples, however legitimate the concerns, is a dangerous trend as it can place global health at risk. In response, global health governance aimed to ‘shift to a stance that placed greater emphasis on disease as a key security threat’ ( Youde, 2019). The initial response adopted of disregarding considerations of sovereignty and emphasizing the importance of a collective global response failed to be effective, as sample and data donor countries were not ‘rewarded for their contributions to the global health initiatives’ ( Carter, 2010), and failed to provide mechanisms that would allow equitable distribution of benefits derived from data and sample sharing.

Looking at the case studies discussed above, three broad principles that form the basis of an ideal model of good governance for sharing samples and data internationally can be identified.

1. Collaboration: The model should recognise that collaboration among all researchers is essential for ethical, fair and sustainable data and sample sharing. Leaving aside ‘parachute research’, which is now increasingly condemned, even beneficence as an underlying consideration, though it has positive connotations, fails to recognise the contributions from the intended beneficiary, preventing true partnership among international colleagues. The H3 Africa initiative, for example, recognised this factor when it termed that the capacity building work done was ‘in return’ for providing access to African data for secondary analysis, rather than as a beneficent measure ( H3Africa consortium, 2018).
In the same vein, research participants in LMICs should also be considered collaborative partners in the research process, as they bear the most significant risks, for example, by placing at risk their privacy and right to confidentiality when providing data and samples. Respect for participants and their communities needs to be displayed through implementing consent measures and adequate protections, and greater efforts need to be taken to propagate patient and public involvement and engagement measures in LMICs, similar to what is being done in the global north.
Funding agencies such as the Wellcome Trust and the MRC, UK, have taken great steps forward in promoting a collaborative approach; they now require collaborations with research institutions in the country where the research is carried out as a pre-condition of funding global health research. This collaborative approach is, however, taking time to dwindle down to other HIC research institutions, which, in some cases claim all rights to intellectual property created through the research collaboration, contrary to the word and intent of the terms and conditions imposed by the funding agency, which specify that ownership of intellectual property lies with the institution generating it.
2. Fairness: Fairness towards all participants is essential for long-term collaborations. A governance framework should ensure fair sharing of benefits with data and sample providers, ensure fairness towards the primary data/sample collecting researchers by recognising their contributions and equally balance the interests of science and the funders of data by ensuring that maximum benefit is derived from the data collected. Simple measures such as a requirement of at least one author from the country where the data is collected, and a ‘taxonomy of contributor-ship’ indicating the roles of authors could help promote fairness ( Allen *et al.*, 2014).
3. Work towards a level playing field: Neither fairness nor collaboration can be effectively achieved with the significant power differential that exists between institutions of the global north and the global south. Many guidelines for good practice in collaborations exist; however, inequity persists. The degree of the inequity depends on the ‘experience and empowerment of the LMIC researchers, research infrastructure… and the amount of funding available’ ( Hedt-Gauthier *et al.*, 2018). For over thirty years (if not more), concerned individuals and institutions from both sides of the divide have been discussing the need for collaboration and capacity building to bridge this gap and over the past few years, signs of progress are visible. Funding agencies such as the Wellcome Trust and the MRC, UK, consider collaborations and capacity building as a factor when evaluating a research funding application, compelling researchers to engage in such efforts.
Human capacity building in LMICs is possibly the most important step to be taken to reach a more level playing field. Lack of technical and research expertise in LMIC research institutions is often cited as the reason for the need for analysis to be carried out in an HIC during an international collaboration. It is time to lay this poor excuse to rest, particularly in data analysis. The world’s most advanced industries using state-of-the-art, high-end technology carry out data analysis functions in LMICs: Google, for example, has nine offices in the South East Asia region, with over 2,000 data scientists / analysts working in India alone. Other ‘big data’ companies – Microsoft, Amazon, Oracle – all run offices in LMICs, having successfully harnessed the human capacity in LMICs. The capacity for data analysis already exists in LMICs, it merely needs to be fine-tuned for health research.

The call for more ethical international data sharing and data governance, as well as for equitable global research collaborations, has been strengthening consistently. Serwadda *et al.* (2018) addressed similar concerns, stating that ‘credit, capacity and engagement’ are necessary for ethical data sharing. Hedt-Gauthier *et al.* (2018), along with colleagues in both Africa and the Americas, recommended steps that could facilitate equity in global health research collaborations, which are also directly applicable to promoting greater equity in data governance frameworks, including: (i) embedding, where HIC collaborators spend enough time in the LMICs of their research to support capacity building, training and mentoring; (ii) rewarding commitment to building long term partnerships, capacity building and ensuring fair research fund sharing with LMIC institutions.

Learning from past experience, developing and implementing ethical governance frameworks so that the interests of both data providers and data users are protected and adopting processes and mechanisms that help promote data sharing but avoid the ‘pitfalls of colonial science’ ( Serwadda *et al.*, 2018) are necessary to build the mutual trust that is critical to continued international data and sample sharing.

## Data availability

No data are associated with this article.
