# Supplementary material for: Advancing good governance in data sharing and biobanking - international aspects
Source: Wellcome Open Res. 2019 Nov 22;4:184. [Version 1] doi: 10.12688/wellcomeopenres.15540.1 (PMC6945104; doi:10.12688/wellcomeopenres.15540.1)
Supplement: Peer review history

# Peer review: Reviewer response for version 1

**DOI:** 10.21956/wellcomeopenres.17014.r37153

**Published:** 2020-01-06

**Authors:** Ravi Vaswani

**Affiliations:** 1 Internal Medicine & Additional Faculty, Centre for Ethics, Yenepoya University, Mangalore, Karnataka, India

Data is the new-age currency in the fiercely market-driven economy of the twenty-first century. Data analysis using information technology has always been an attractive proposition, but in the modern era of social media and crowd-sourcing, artificial intelligence is fast replacing the conventional IT algorithms. In such a rapidly evolving global scenario, this article which uses case studies to bring to the fore, the lack of an the ethically sound "good governance" framework to guide international research collaborations, is very timely, relevant and welcome. 

 The authors have reviewed the existing literature extensively and have referenced many texts addressing various issues relating to the development of a good governance framework that will ethically satisfy all research stakeholders. 

 The use of 2 case studies from different LMICs has provided a balanced and unbiased opinion on the current situation as well as the recommendations for the core requirements in a good governance framework. 

 Two issues missing from the analysis include: 

1. The fact that research is driven by market forces and not by academic pursuit. This necessitates looking at data as an equivalent of bitcoin, since the bottom line for all market-oriented companies is profit. How this influences the way data from LMICs is used by HICs needs to be addressed.
2. Besides the power inequities mentioned by the authors as a reason for unfairness in data-sharing, another angle that needs consideration is the notion of widespread corruption in LMICs. If data is a commodity to be bought and sold then it is tempting to consider that "poor" nations might "sell" the data of their citizens to the "rich" corporate bodies that are believed to be controlling or directing the course of research, for personal or organizational gains. How much of an impact does societal corruption have to play in the issue of data-sharing and how it can be prevented from influencing how data-sharing is done could be factored into the framework suggested. If not the framework, in the introduction or the issues at hand it could deserve a mention. 

I confirm that I have read this submission and believe that I have an appropriate level of expertise to confirm that it is of an acceptable scientific standard.

# Peer review: Reviewer response for version 1

**DOI:** 10.21956/wellcomeopenres.17014.r37150

**Published:** 2019-12-16

**Authors:** Chandanie Wanigatunga

**Affiliations:** 1 Department of Pharmacology, Faculty of Medical Sciences, University of Sri Jayewardenepura, Nugegoda, Sri Lanka; 2 Ethics Review Committee, Sri Lanka Medical Association, Colombo, Sri Lanka

Over all, a good paper which details the issues faced by LMIC with regard to data sharing. The two case reports are well presented and issues correctly analysed to highlight the issues.

 An issue that would make the article difficult to read is the use of very long sentences. In some instances, a single sentence constitutes a complete paragraph (para 2 in the background section). This is seen mainly in the background section and in case study 1. The sentences are much shorter in case study 2, but here too there are instances where long sentences have been used.

 I would strongly recommend that the manuscript be reviewed by the authors with a view to using shorter sentences wherever possible.

 Citations are needed for the following:

 - **Case study 1**:

  - Research ethics from a developing world perspective 

 - to substantiate statement that  "IRD helped build expertise not only in Sri Lanka, but the entire South Asian region". 

 - The statement "For example, the case studies usually provided are rarely applicable in the developing world context..." should be supported with evidence. It would also be useful to briefly state the issues identified that are not relevant to LMIC.

I confirm that I have read this submission and believe that I have an appropriate level of expertise to confirm that it is of an acceptable scientific standard.
